# Genetic Characterization and Classification of Human and Animal Sapoviruses

**DOI:** 10.1371/journal.pone.0156373

**Published:** 2016-05-26

**Authors:** Tomoichiro Oka, Zhongyan Lu, Tung Phan, Eric L. Delwart, Linda J. Saif, Qiuhong Wang

**Affiliations:** 1 Food Animal Health Research Program, Department of Veterinary Preventive Medicine, Ohio Agricultural Research and Development Center, College of Food Agriculture and Environmental Sciences, The Ohio State University, Wooster, OH, United States of America; 2 Department of Virology II, National Institute of Infectious Diseases, Musashi-murayama, Tokyo, Japan; 3 Blood System Research Institute, 270 Masonic Avenue, San Francisco, CA, United States of America; 4 University of California San Francisco, Dept of Laboratory Medicine, San Francisco, CA, United States of America; University of Hong Kong, HONG KONG

## Abstract

Sapoviruses (SaVs) are enteric caliciviruses that have been detected in multiple mammalian species, including humans, pigs, mink, dogs, sea lions, chimpanzees, and rats. They show a high level of diversity. A SaV genome commonly encodes seven nonstructural proteins (NSs), including the RNA polymerase protein NS7, and two structural proteins (VP1 and VP2). We classified human and animal SaVs into 15 genogroups (G) based on available VP1 sequences, including three newly characterized genomes from this study. We sequenced the full length genomes of one new genogroup V (GV), one GVII and one GVIII porcine SaV using long range RT-PCR including newly designed forward primers located in the conserved motifs of the putative NS3, and also 5' RACE methods. We also determined the 5’- and 3’-ends of sea lion GV SaV and canine GXIII SaV. Although the complete genomic sequences of GIX-GXII, and GXV SaVs are unavailable, common features of SaV genomes include: 1) “GTG” at the 5′-end of the genome, and a short (9~14 nt) 5**′**-untranslated region; and 2) the first five amino acids (M [A/V] S [K/R] P) of the putative NS1 and the five amino acids (FEMEG) surrounding the putative cleavage site between NS7 and VP1 were conserved among the chimpanzee, two of five genogroups of pig (GV and GVIII), sea lion, canine, and human SaVs. In contrast, these two amino acid motifs were clearly different in three genogroups of porcine (GIII, GVI and GVII), and bat SaVs. Our results suggest that several animal SaVs have genetic similarities to human SaVs. However, the ability of SaVs to be transmitted between humans and animals is uncertain.

## Introduction

Sapoviruses (SaVs), members of the *Sapovirus* genus within the *Caliciviridae* family, have been detected from humans [1] and animals (pigs [2, 3], mink [4], dogs [5], sea lions [6], bats [7], chimpanzees [8], and rats [9], listed in the order of their discovery). The SaV genome is a linear, positive sense, single stranded RNA of 7.1–7.7 kb in size with a poly(A) tail at the 3’-end. It is predicted to encode viral nonstructural proteins (NSs) (NS1, NS2, putative NTPase [NS3], NS4, VPg [NS5], and fused protease–RNA-dependent RNA polymerase [RdRp] [NS6-NS7]), and structural proteins VP1 and VP2 [1]. SaVs are genetically highly diverse and currently classified into 14 genogroups (G) based on the complete VP1 amino acid sequences [10]. Typical conserved amino acid sequences were identified in the NS3 (GAPGIGKT), NS5 (KGKTK and DDEYDE), NS6 (GDCG), NS7 (WKGL, KDEL, DYSKWDST, GLPSG, and YGDD), and VP1 (PPG and GWS) among the GI-GV SaVs [1].

SaVs have been detected from pigs (GIII, GV-GXI), mink (GXII), and bats (GXIV), using primers targeting the NS7 region [2, 4, 7, 10–15]. In addition, SaV sequences have been detected from dogs (GXIII) and sea lions (GV) by next generation sequencing [5, 6]. A variety of SaVs were also detected from humans using primers targeting the NS7 or VP1 region, or by next generation sequencing [1, 16]. Current animal and human SaVs were classified into 11 genogroups (GIII, and GV–GXIV) and four genogroups (GI, GII, GIV, and GV), respectively, based on the complete VP1 sequences [10]. GV SaVs are detected from both animals and humans [1].

At the beginning of this study, there were 28 complete sapovirus genomes available in the literature or Genbank database: GI (n = 9), GII (n = 5), GIV (n = 4), and GV (n = 3) from humans; GIII (n = 3), GVI (n = 2), and GVII (n = 1) from swine; and GXIV (n = 1) from bats. In addition, the nearly full length genomic sequences, excluding the 5’- and 3’-ends, of GV sea lion and GXIII canine SaVs were reported [5, 6].

During our investigation, new SaVs were detected from chimpanzees [8] and rats [9] by next generation sequencing. The nearly full length genomic sequences, excluding the 5’- and 3’-ends, of the chimpanzee SaVs have been determined and are classified as GI based on the complete VP1 sequences. The VP1 sequences of rat SaVs have been determined, but they are not yet classified [9]. Furthermore, one more complete genome sequence of GIII porcine SaV (CH430 strain) has been determined [17].

Available complete genome sequences for animal SaVs are still limited compared to human SaVs. Therefore, the aims of this study were to determine additional complete genomic sequences of animal SaVs, to identify the common genetic characteristics of SaVs, and to examine the genetic relatedness among human and animal SaVs. We also proposed 15 genogroups for human and animal SaVs based on the complete VP1 sequences.

## Materials and Methods

### New forward primers designed for the amplification of swine and mink SaVs by RT-PCR

The genomic sequences of seven animal SaVs [3 GIII (Genbank accession no. AF182760, FJ387164, and JX678943), 2 GVI (AY974192 and KJ508818), 1 GVII (AB221130) and 1 GXIV (JN899075)] were aligned using ClustalW version 2.1 (http://clustalw.ddbj.nig.ac.jp/top-j.html). New primers, 1550F [5**′**- CCBTDMCCAYTGRAYTGTGA-3**′**] and 1571F [5**′**- CCCTWMCCAYTGAATTGTGA-3**′**] targeting the conserved “PL (N / D) CD” sequence, and 1578F [5**′**- TGGGACGAGTTTGACAC -3**′**] targeting “WDEFD” sequence, were designed and synthesized at Integrated DNA Technologies, Inc.

### Fecal specimens

Swine, mink, dog, and sea lion fecal samples from previous studies [4–6, 10, 18–20] were stored at -80°C and were used in this study for further sequence analyses. SaVs of swine fecal origin used in this study (GVII WGP247 [KC309421]; GVIII WG194D [KC309416], WG197C [KC309417], and WG214D [KC309419]; GIX WG214C [KC309418]) had been determined previously for the 3’-end ~ 3kb fragment, covering from the partial NS7 to the 3’-end of the genome. Mink SaV sequence had been determined for only partial NS7 sequence using mixed samples of five feces [4]. The nearly complete genomic sequences of sea lion SaV (GV/CSL9775 [Genbank accession no. JN420370]) and dog SaV (GXII/AN210D [JN387134]) fecal origin had been de novo assembled using next generation sequencing data [5, 6]. We included them in this study because the 5**′** and 3**′** ends of the sea lion SaV, and the 5**′** end of dog SaV were lacking based on comparisons to other SaV full genomes using The Basic Local Alignment Search Tool (BLAST http://www.ncbi.nlm.nih.gov/BLAST).

### RNA extraction, cDNA synthesis, and PCR

Viral RNA was extracted from 200 μL of fecal suspensions using RNeasy Mini kit (Qiagen) with slight modification. Briefly, 200 μL of fecal suspensions was mixed with 350 μL of RLT buffer and incubate for 5 min, then 296 μL of ethanol was added and mixed well. The mixture was applied onto RNeasy mini column and then washed and eluted according to the manufacture’s instructions. Purified RNA was eluted in 40 μL UltraPure DNase/RNase-Free Distilled Water (Invitrogen) and used freshly or stored at -80°C.

cDNA was synthesized as follows: 8.5 μL of viral RNA solution was mixed with 0.5 μL of the 10 pmol/μl reverse primer [TX30SXN (5**′**- GACTAGTTCTAGATCGCGAGCGGCCGC CCT_30_−3**′**) [21] that was complementary to the 3’-end including partial polyA tail or the gene-specific reverse primer targeting the NS7 and VP1 junction region], and 1 μL of 10 mM dNTP. The mixtures were incubated at 65°C for 5 min, cooled on ice, and then mixed with 2 μL of 10 × First strand buffer (Invitrogen), 2 μL of 100 mM DTT (Invitrogen), 4 μL of 25 mM MgCl_2_ (Invitrogen), 1 μL of RNase OUT ribonuclease inhibitor (40 U/μl) (Invitrogen), and 1 μL of SuperScript III reverse transcriptase (200 U/μl) (Invitrogen). This mixture was incubated first at 25°C for 5 min, then at 50°C for 50 min, and finally was inactivated at 95°C for 5 min.Next, 1 μL of RNase H (2 U/μl) (Invitrogen) was added and incubated at 37°C for 20min.

The SaV genomic sequences spanning the putative NS3 to NS7 region or the putative NS3 to the end of the genome were amplified by RT-PCR with one of the newly designed forward primers (1550F, 1571F, or 1578F) and reverse primer TX30SXN or the strain-specific reverse primer targeting the NS7 -VP1 junction region, using high-fidelity PCR enzyme, PrimeSTAR GXL DNA polymerase (TaKaRa Mirus Bio). A final volume of 50 μ l of the PCR reaction mixture contained 2 μL of the cDNA or the first PCR products, 10 μ l of 5 × PrimeSTAR GXL DNA polymerase buffer, 4 μ l of 2.5 mM dNTPs, 2 μ l of forward primer (10 pmol/μ l), 2 μ l of reverse primers (10 pmol/μ l), and 1 μL of PrimeSTAR GXL DNA polymerase (1.25 U/μ l). PCR was performed at 98°C for 10 sec followed by 45 cycles of 98°C for 10 s, 55°C for 15 s, and 72°C for 2 min, and a final extension at 72°C for 10 min.

For the new porcine GV SaV, the sequence covering the partial NS3 to the 3’-end of the genome was amplified by semi-nested RT-PCR using gene-specific forward primers and TX30SXN primer, as described above.

### 5′ RACE

The 5′ terminal nucleotide sequences of the SaV genomes were determined using 5' Rapid Amplification of cDNA Ends (RACE), Version 2.0 (Invitrogen) with a slight modification from the original protocol. Briefly, cDNA was synthesized from RNA as follows: 8.5 μL of viral RNA was mixed with 0.5 μL of 10 pmol/μl strain-specific primer and 1 μL of 10 mM dNTPs. The mixture was incubated at 80°C for 3 min, cooled on ice, and then mixed with 2 μL of 10 × First strand buffer, 2 μL of 100 mM DTT, 4 μL of 25 mM MgCl_2_, 1 μL of RNase OUT ribonuclease inhibitor (40 U/μl), and 1 μL of SuperScript III reverse transcriptase (200 U/μl). This mixture was incubated first at 25°C for 5min, then at 50°C for ~3h, and finally at 85°C for 5 min. Afterwards, 1 μ l of RNase H or RNase T1 mixture (Invitrogen) was added to the mixture that was incubated at 37°C for 30 min. The cDNA was purified using the SNAP column in the 5' RACE System (Invitrogen) or the column in the QIAGEN PCR purification kit (Qiagen) according to the manufacture’s instructions. Finally, the purified cDNA was eluted in 50 μL of UltraPure DNase/RNase-Free Distilled Water. Homopolymeric (dC or dA) tailing was added on the purified cDNA as follows: 10 μL of the purified cDNA solution, 2.5 μL of 2.5mM dATP (promega) or dCTP (Invitrogen), 5 μL of 5 x tailing buffer, and 6.5 μL of water was mixed, incubated at 94°C for 3 min, and cooled on ice.Then 1 μL of Terminal deoxynucleotidyl transferase (20 U/μl) (Invitrogen) was added and the mixture was incubated first at 37°C for 10min and then at 65°C for 10 min to inactivate the enzyme. Nested PCRs were performed with the gene specific reverse primers and the abridged anchor primer AAP (5′-GGCCACGCGTCGACTAGTACGGGIIGGGIIGGGIIG-3′) for the primary PCR and the abridged universal primer AUAP (5′-GGCCACGCGTCGACTAGTAC-3′) (Invitrogen) for the secondary PCR for the poly (C)-tailed cDNA, and QT (5′- CCAGTGAGCAGAGTGACGAGGACTCGAGCTCAAGCT_17_-3′) and QO (5′- CCAGTGAGCAGAGTGACG-3′)for poly (A)-tailed cDNA, using high-fidelity PCR enzyme, PrimeSTAR GXL DNA polymerase or PrimeSTAR HS DNA polymerase (TaKaRa Mirus Bio). A final volume of 50 μ l of the reaction mixture contained 5 μL of the homopolymeric (dC or dA) tailing cDNA or the 0.2 μL of the first PCR reaction mixture, 10 μ l of 5 × PrimeSTAR DNA polymerase buffer, 4 μ l of 2.5 mM dNTPs, 2 μ l of forward primer (10 pmol/μ l), 2 μ l of reverse primer (10 pmol/μl), and 1 μL of PrimeSTAR HS DNA polymerase (2.5 U/μl) or PrimeSTAR GXL DNA polymerase (1.25 U/μ l). PCR was performed at 95°C for 5 min for initial denaturing, followed by 45 cycles of 94°C for 15 s, 60°C for 15 s, and 72°C for 2 min, and a final extension at 72°C for 10 min.

### Amplification of the 3′ ends of the SaV genomes

The 3**′** end of a SaV genome was amplified by RT-PCR with gene specific forward primer, and the reverse primer TX30SXN, using high-fidelity PCR enzyme. A final volume of 50 μl of the reaction mixture contained 2 μL of the cDNA synthesized with the TX30SXN primer, 10 μl of 5 × PrimeSTAR DNA polymerase buffer, 4 μ l of 2.5 mM dNTPs, 2 μ l of forward primer (10 pmol/μ l), 2 μ l of reverse primer (10 pmol/μ l), and 1 μL of PrimeSTAR HS DNA polymerase (2.5 U/μ l) or PrimeSTAR GXL DNA polymerase (1.25 U/μ l). PCR was performed at 95°C for 5 min for initial denaturing followed by 35 cycles of 98°C for 10 s, 55°C for 15 s, and 72°C for 1 min, and a final extension at 72°C for 10 min.

### Cloning, sequencing, phylogenetic analyses, and genogrouping

The PCR products were separated by agarose gel electrophoresis, purified using a QIAquick Gel Extraction Kit (Qiagen), and sequenced directly or cloned into the pCR4Blunt-TOPO vector (Invitrogen) before sequencing by primer walking methods using a set of gene-specific primers. For cloned samples, at least three positive clones of each sample were selected for sequencing. Samples were sequenced using BigDye Terminator cycle chemistry and an automated ABI Prism3100xl sequencer (Applied Biosystems). Sequence editing and assembly were performed using the Sequencher program v4.10.1 (GeneCodes) and analyzed by Genetyx-Mac software v16.0.4 (Genetyx Corporation). The Basic Local Alignment Search Tool (BLAST; http://blast.ncbi.nlm.nih.gov) was employed to find homologous hits. Amino acid sequences were aligned using ClustalW version 2.1 (http://clustalw.ddbj.nig.ac.jp/top-j.html). The construction of Maximum-likelihood phylogenetic trees with 1,000 bootstrap replications, and the calculation of amino acid sequence pairwise distances were performed using MEGA6 software [22]. Identity = 1—distance.

### Nucleotide sequence accession numbers

The SaV nucleotide sequences determined in this study have been deposited in GenBank under accession numbers KX000383 for GV/WG194D-1, KX000384 for GVII/RV0042, and KX000385 for GXII/WD1237. The nucleotide sequences of GV/CSL9775 (JN420370), GVII/WGP247 (KC309421), GVIII/WG214D (KC309419), GVIII/WG194D (KC309416), GVIII/WG197C (KC309417), GIX/214C (KC309418), and GXIII/AN210D (JN387134) SaVs have been updated in GenBank database.

## Results and Discussion

### Forward Primers targeting the putative NS3 region were designed to amplify SaVs from different animal species

**Fig 1:**
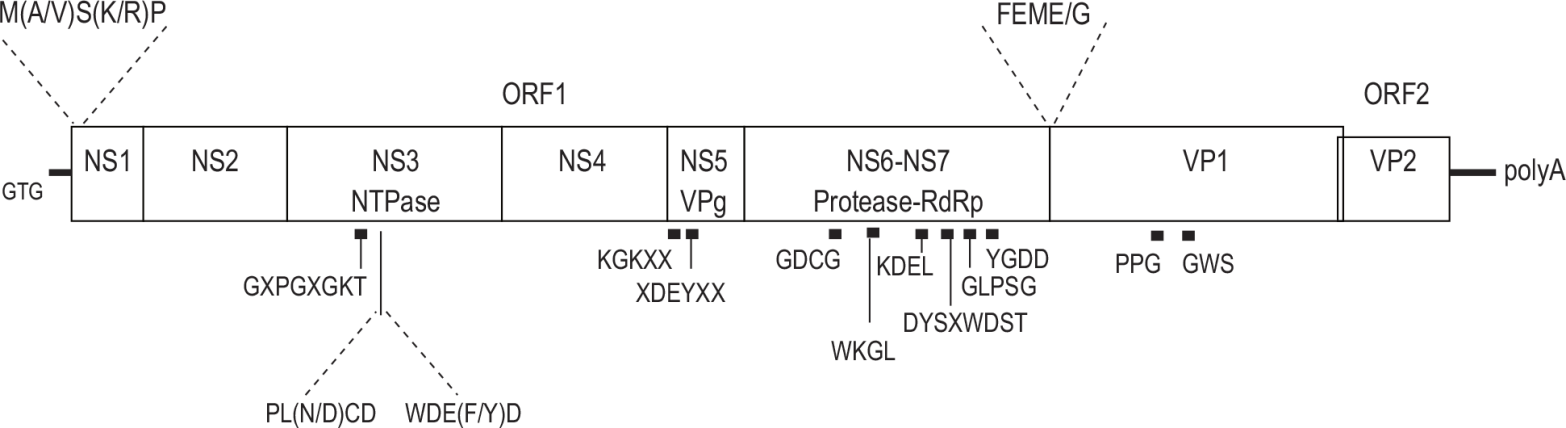
Sapovirus genomic organization: non-structural (NS) and structural proteins VP1 and VP2, and the conserved motifs. Genomic organization of a common sapovirus, including two open reading frames (ORF1 and ORF2). ORF1 encodes the predicted viral NS proteins (NS1, NS2, NS3 [NTPase], NS4, NS5 [VPg], NS6-NS7 [protease-RNA-dependent RNA polymerase (RdRp)], and the major capsid protein (VP1). ORF2 encodes the minor capsid protein VP2. The following amino acid or nucleotide motifs are conserved among all available sequenced SaVs: GXPGXGKT, PL (N / D) CD, and WDE (F / Y) D of NS3, KGKXX and XDEYXX of NS5, GDCG of NS6, WKGL, KDEL, DYSXWDST, GLPSG, and YGDD of NS7, PPG and WGS of VP1, and the first three nucleotides (GTG) at the 5’–end. Also shown are the first five amino acids of NS1 (M [A / V] S [K / R] P) and around the NS7-VP1 cleavage site (FEME / G, the slash indicates the putative cleavage site by viral protease NS6) that are conserved among GI-GV, GVIII, and GXIII SaVs.

We found that the regions suitable for PCR primer design were in the putative NS3 region based on the full genomic sequence alignments of the seven animal SaVs. We designed three forward primers, 1550F, 1571F, and 1578F (see Materials and Methods). The primers 1550F and 1571F targeted the same PL (N / D) CD amino acid motif that was conserved among GIII, GVI, GVII, and GXIV SaVs, and the primer 1578F targeted the WDEFD amino acid motif of GXIV SaVs. This motif (WDEYD) differed slightly in GVI and GVII SaVs. These motifs were located downstream of the typical GXPGXGKT motif of the putative NS3 (Fig 1).

### Successful amplification of GV, GVII, GVIII, and GXII SaVs, but not GIX SaVs, using the newly designed forward primers

We amplified the sequence fragments for two porcine GVII (RV0042 and WGP247), three porcine GVIII (WG214D, WG194D and WG197C), and one mink GXII (WD1237]) SaVs using the newly designed forward primers 1550F, 1571F, or 1578F and strain-specific reverse primers or the modified oligo dT primer, TX30SXN. The strain specific reverse primer for WD1237 was designed based on the partial sequence obtained by next generation sequence (NGS) as described previously (Li et al., 2011).

A long PCR product (approximately 6kb) was amplified for SaV strain RV0042 using primer set 1571F and TX30SXN. Approximately 3.5 kb-fragments were amplified for other samples using these three forward primer(s) and the corresponding strain-specific reverse primers targeting the NS7-VP1 junction region.

We could not amplify the fragment from GIX SaV (WG214C) using those forward primers. In most successful cases, only one of the three forward primers (1550F, 1571F, or 1578F) amplified a SaV strain, except for GVIII WG194D and WG197C samples. Two different SaVs, GVIII (WG194D) and GV SaVs (WG194D-1) were amplified from the same fecal sample WG194D using 1550F and 1578F primers, respectively. Identical sequences were amplified for WG197C strain using 1550F and 1571F forward primers.

### The complete full length genomes of GV, GVII, GVIII, GXIII SaVs were determined using RACE methods

**Fig 2:**
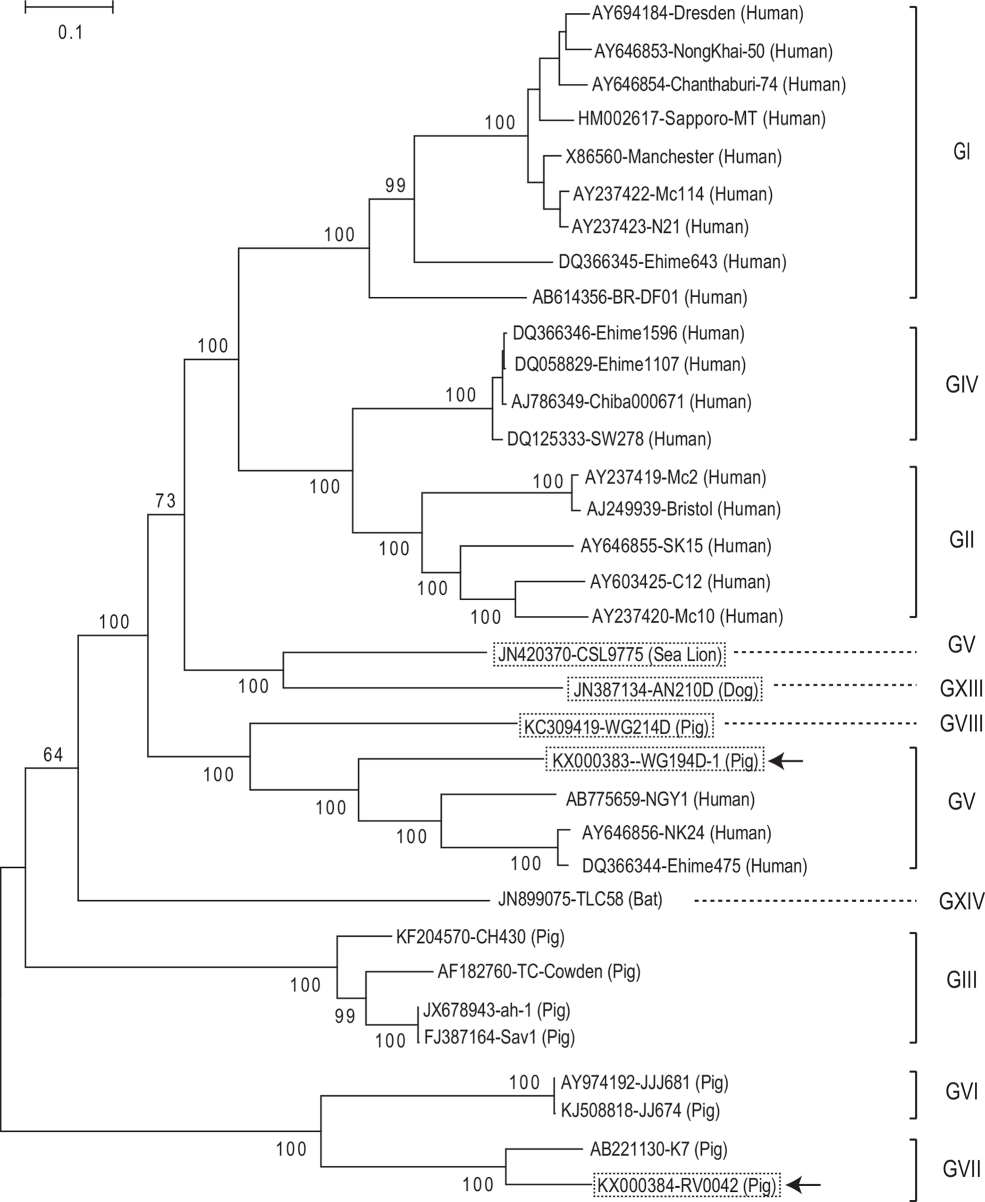
Phylogenetic tree of the full length genomic sequences of 34 sapovirus strains using MEGA 6. The five strains whose complete genomes were determined in this study are boxed with dotted lines. Among these, the two SaV strains that were newly identified in this study are also indicated with arrows. The number on each branch indicates the bootstrap value. The scale represents the amino acid substitutions per site. Each sapovirus strain is indicated in the following format: Genbank accession number-strain name (species).

We further performed 5**′**-RACE for the porcine SaVs (GV, GVII, GVIII, and GIX) and mink SaV (GXII). Among the eight SaV strains, we successfully determined the complete genomic sequences of three strains (Po/SaV/GV/WG194D, Po/SaV/GVII/RV0042, and Po/SaV/GVIII/WG214D) (Table 1, Fig 2). The genome of GV/WG194D-1 was 7496 nt long, excluding the poly(A) tail, and shared 68% nt identity with other GV SaV genomes (Genbank accession numbers AB775659, AY646856, and DQ366344). The genome of GVII/RV0042 was 7150 nt long, and it shared 83% nt identity with the GVII/K7 strain (AB221130) detected in Japan. The genome of GVIII/214D strain was 7497 nt long and represented the first complete genome sequence for a porcine GVIII SaV. In this study, we also determined the 5’- and 3’-ends of sea lion/GV/CSL9775 strain (60 nt of 5**′** end and 60 nt of 3**′** end) and Canine/GXIII/AN210D strain (330 nt of 5**′** end), and their genome sizes were 7497 and 7469 nt long, respectively (Table 1, Fig 2).

**Table 1:** Genomic characteristics of 50 representative sapovirus strains in 15 genogroups.

Genogroup	Genbank accession no.	Strain name*	Species	Genome size (nt)**	The 5'-end 30 nt sequences***	Length of 5-UTR (nt)	First five amino acid residues in the NS1	Five amino acid residues at the putative NS7-VP1 cleavage site
GI	X86560	Manchester	Human	7431	gtgattggtt agATGgtttc caagccattc	12	MVSKP	FEMEG
	HM002617	Sapporo-MT	Human	7433	gtgattggtt agATGgtttc caagccattc	12	MVSKP	FEMEG
	AY694184	Dresden	Human	7429	gtgattggtt agATGgtttc caagccattc	12	MVSKP	FEMEG
	AY237422	Mc114	Human	7429	gtgattggtt agATGgcttc caagccattc	12	MASKP	FEMEG
	AY237423	N21	Human	7429	gtgattggtt agATGgcttc caagccattc	12	MASKP	FEMEG
	AY646853	NongKhai-50	Human	7429	gtgattggtt ggATGgcttc caagccattc	12	MASKP	FEMEG
	AY646854	Chanthaburi-74	Human	7429	gtgattggtt ggATGgcttc caagccattc	12	MASKP	FEMEG
	AB614356	BR-DF01	Human	7476	gtgattggtt agATGgtttc caagccatac	12	MVSKP	FEMEG
	DQ366345	Ehime643	Human	7447	gtgattggtt agATGgcttc taagccatat	12	MASKP	FEMEG
	KJ858686	IJC04	Chimpanzee	(7320)	———-—ATGgcttc caagccattc	-	MASKP	FEMEG
GII	AJ249939	Bristol	Human	7490	gtgattggtt ggtATGgctt ctaagccatt	13	MASKP	FEMEG
	AY237419	Mc2	Human	7490	gtgattggtt ggtATGgctt ctaagccatt	13	MASKP	FEMEG
	AY237420	Mc10	Human	7458	gtgattggtt agtATGgctt ccaagccatt	13	MASKP	FEMEG
	AY603425	C12	Human	7476	gtgattggtt ggATGgcttc caagccattc	12	MASKP	FEMEG
	AY646855	SK15	Human	7459	gtgattggtt agtATGgctt ccaagccatt	13	MASKP	FEMEG
	KJ950881	NYC-A1	Rat	(1650)	NA****	-	NA	—MEG
	KJ950882	NYC-B2	Rat	(1650)	NA	-	NA	—MEG
GIII	AF182760	TC-PEC	Pig	7320	gtgatcgtgA TGgctaattg ccgtccgttg	9	MANCR	FVMEA
	AY425671	LL14	Pig	(7291)	———A TGgctaattg ccgtccgtta	-	MANCR	FVMEA
	FJ387164	SaV1	Pig	7541	gtgatcgtgA TGgctaattg ccgtccgtta	9	MANCR	FVMEA
	JX678943	ah-1	Pig	7342	gtgatcgtgA TGgctaattg ccgtccgtta	9	MANCR	FVMEA
	KF204570	CH430	Pig	7341	gtgatcgtgA TGcctaattg ccgtccgttg	9	MPNCR	FVMEA
GIV	AJ786349	Chiba00671	Human	7420	gtgattggtt agtATGgctt ctaagccatt	13	MASKP	FEMEG
	DQ058829	Ehime1107	Human	7427	gtgattggtt agtATGgctt ctaagccatt	13	MASKP	FEMEG
	DQ125333	SW278	Human	7437	gtgattggtt agtATGgctt ctaagccatt	13	MASKP	FEMEG
	DQ366346	Ehime1596	Human	7428	gtgattggtt agtATGgctt ctaagccatt	13	MASKP	FEMEG
GV	AY646856	NK24	Human	7500	gtgatcacct tgggATGgct tccaagccac	14	MASKP	FEMEG
	DQ366344	Ehime475	Human	7500	gtgatcacct tgggATGgct tccaagccac	14	MASKP	FEMEG
	AB775659	NGY-1	Human	7521	gtgatcacct tgggATGgct tccaagccat	14	MASKP	FEMEG
	AB521771	TYMPo239	Pig	(3949)	NA	-	NA	FEMEG
	AB521772	TYMPo31	Pig	(3949)	NA	-	NA	FEMEG
	JN420370	**CSL9775**	Sea lion	7497	gtgattggtt tgcgATGgcc tcaaagccat	14	MASKP	FEMEG
	KX000383	**WG194D-1**	Pig	7496	gtgatcactt tgagATGgct tcaaagccat	14	MASKP	FEMEG
GVI	AY974192	JJ681	Pig	7198	gtgtatagtt ATGgcggcta cttgccgtca	10	MAATC	YTMEG
	KJ508818	JJ674	Pig	7198	gtgtatagtt ATGgcggcta cttgccgtca	10	MAATC	YTMEG
GVII	AB221130	K7	Pig	7144	gtgaacgttA TGgcggctac ttgccgtcat	9	MAATC	YKMEG
	KX000384	**RV0042**	Pig	7150	gtgaacgttA TGgcggctgt ttgccgtcat	9	MAAVC	YKMEG
	KC309421	**WGP247**	Pig	(6052)	NA	-	NA	YVMEG
GVIII	KC309419	**WG214D**	Pig	7497	gtgatagctt tgATGgcctc ccggcctttc	12	MASRP	FEMEG
	KC309416	**WG194D**	Pig	(6654)	NA	-	NA	FEMEG
	KC309417	**WG197C**	Pig	(6497)	NA	-	NA	FEMEG
GIX	KC309418	**WG214C**	Pig	(3695)	NA	-	NA	YVMEG
GX	AB242873	K8	Pig	(1617)	NA	-	NA	—MEG
GXI	DQ359100	2053P4	Pig	(1635)	NA	-	NA	—MEG
GXII	KX000385	**WD1237**	Mink	(5816)	NA	-	NA	FEMEG
	AY144337	Canada 151A	Mink	(4556)	NA	-	NA	FEMEG
GXIII	JN387134	**AN210D**	Dog	7469	gtgatttgtt gttATGgctt ccaagccatt	13	MASKP	FEMEG
GXIV	JN899075	TLC58	Bat	7695	gtgatagtgA TGgcggcgtt aagccgtgtg	9	MAALS	FVMEG
GXV	KJ950878	NYC-A19	Rat	(1653)	not available	-	NA	—MEG
	KJ950880	NYC-E48	Rat	(1653)	not available	-	NA	—MEG

*The strains whose complete or partial genomic sequence were determined in this study are bolded.

** The complete genomic size does not exclude poly(A) tail, and the strains whose complete genomic sequence are not available yet are indicated by parentheses.

*** The predicted start codon is shown as capital letters.

****NA: Not available

The 5**′**-end of the newly determined GV, GVII, GVIII, and GXIII SaVs started with “GTG”, which was the same as for the other SaV strains (Table 1). The 5**′**- untranslated regions were 9 to 14 nt long, which shared the same size as other SaVs (Table 1).

We also determined the partial genomic sequences covering from the amino acid sequence GXPGXGKT in the putative NS3 region to the 3’-end of Po/GVII/WGP247 (6052nt), Po/GVIII/WG194D (6654nt) and Po/GVIII/WG197C (6497nt) strains, and covering amino acid sequence PLNCD in the putative NS3 region to the 3’- end of Mink/GXII/WD1237 (5816nt) strain, and covering from the amino acid sequence WKGL in the putative NS7 region to the 3’- end of Po/GIX/214C (3695nt) strain (Table 1 and Fig 1). These newly determined complete and partial SaV genomic sequences had typical conserved amino acid motifs for NS5 (KGKXX and XDEYXX), NS6 (GDCG), NS7 (WKGL, KDEL, DYSXWDST, GLPSG, and YGDD), and VP1 (PPG and WGS).

### Genetic comparisons among animal and human SaVs

**Fig 3:**
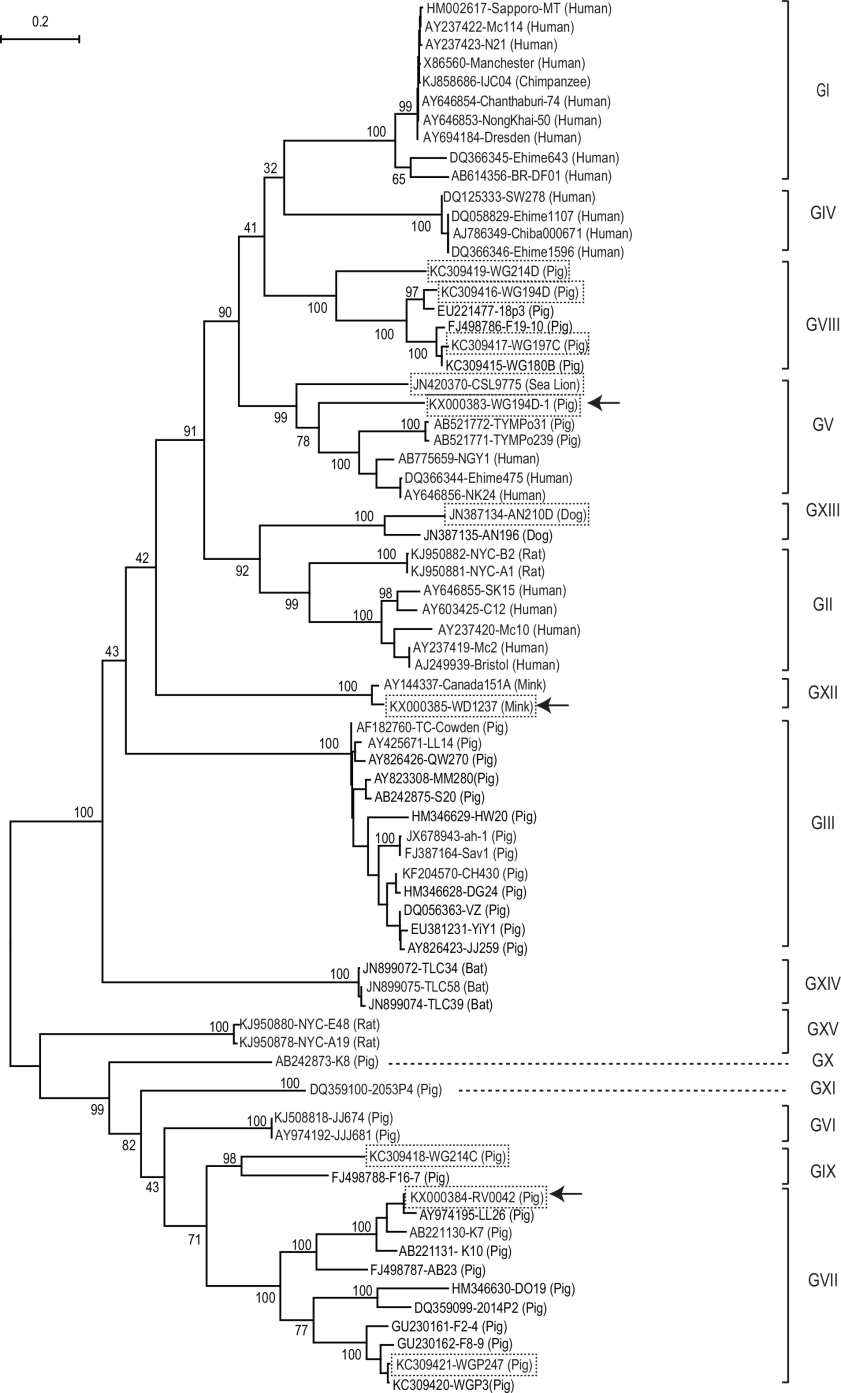
Phylogenetic tree based on the complete VP1 amino acid sequences of 74 sapovirus strains. These strains represent all reported 14 genogroups (GI-GXIV) and the newly reported rat SaV strains using MEGA 6. The 10 animal SaV strains with additional sequences determined in this study are boxed with dotted lines. Among these, the three SaV strains that were newly identified in this study are also indicated with arrows. The number on each branch indicates the bootstrap value. The scale represents the amino acid substitutions per site. Each sapovirus strain is indicated in the following format: Genbank accession number-strain name (species).

The two porcine SaVs (WG194D-1 and RV0042), and the mink SaV (WD1237) clustered with GV, GVII, and GXII SaV strains, respectively, based on phylogenetic analysis of the complete VP1 amino acid sequences (Fig 3).

**Fig 4:**
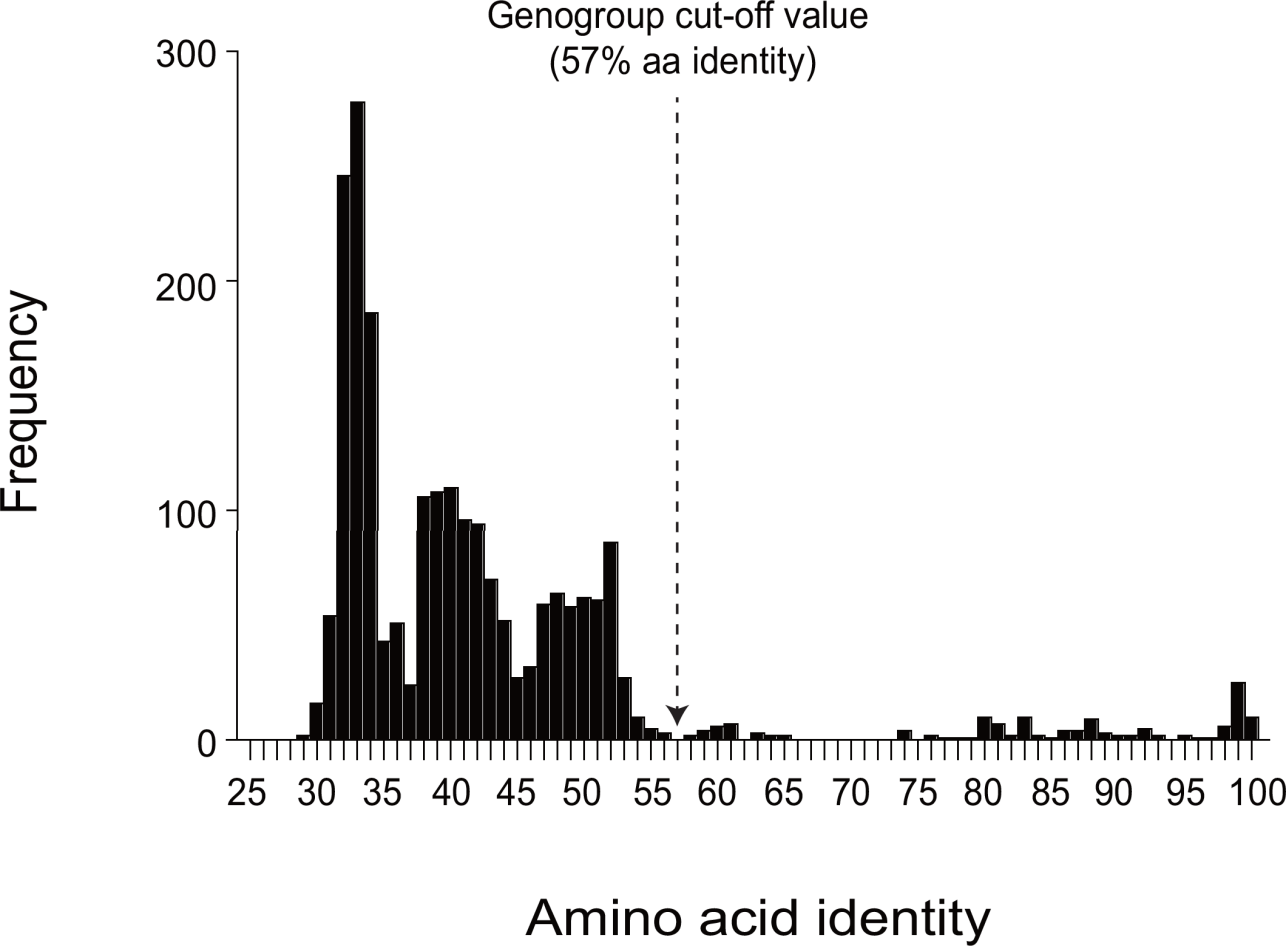
Identity distribution of the VP1 amino acid sequences of 74 SaV strains. The arrow indicates the genogrouping cut-off (≥ 57% aa identity) used in this study.The genogrouping of GV, GVII, GVIII, GIX, GXII, and GXIII SaVs sequenced in this study (as indicated as dotted box in Figs 3 and 5) based on NS7 and VP1 matched, except for the sea lion GV/CSL9775 and the porcine GVII/WGP247 strains. The Sea lion/GV/CSL9775 strain clustered together with other human and porcine GV SaV strains in the VP1 region, but it was separated from other GV strains in the NS7 region as recently discussed [1]. Similarly, GVII/ WGP247 strain clustered together with other GVII strains in the VP1 region, but it was closer to porcine GIX/WG214C in the NS7 region. The NS7 sequences of porcine GX and GXI SaVs and rat (GII and GXV) SaVs are not yet available (Table 1).

The SaVs detected from rat formed two distinct clusters (NYC-A19 and E48 cluster, and NYC-A1 and B2 cluster) based on the VP1 aa sequence similarity (Fig 3), as reported recently [9]. We proposed NYC-A19 and E48 as a new genogroup GXV, because they shared only 29.4–41.8% aa identity to other genogroups of SaVs. We also proposed NYC-A1 and B2 strains as GII, because they shared 59.6–61.1% aa identity with other human GII SaV strains. Compared with other genogroups, lower intra-genogroup aa identity was observed for GV (≥ 57.5%) and GVII (≥ 57.3%) SaV strains. We adjusted the previously proposed cut-off value of 60% VP1 aa identity for genogrouping [10] to a slightly lower value (57% identity or 43% distance) based on phylogenetic and amino acid sequence identity analysis with the newly available SaV sequence data (Figs 3 and 4).

**Fig 5:**
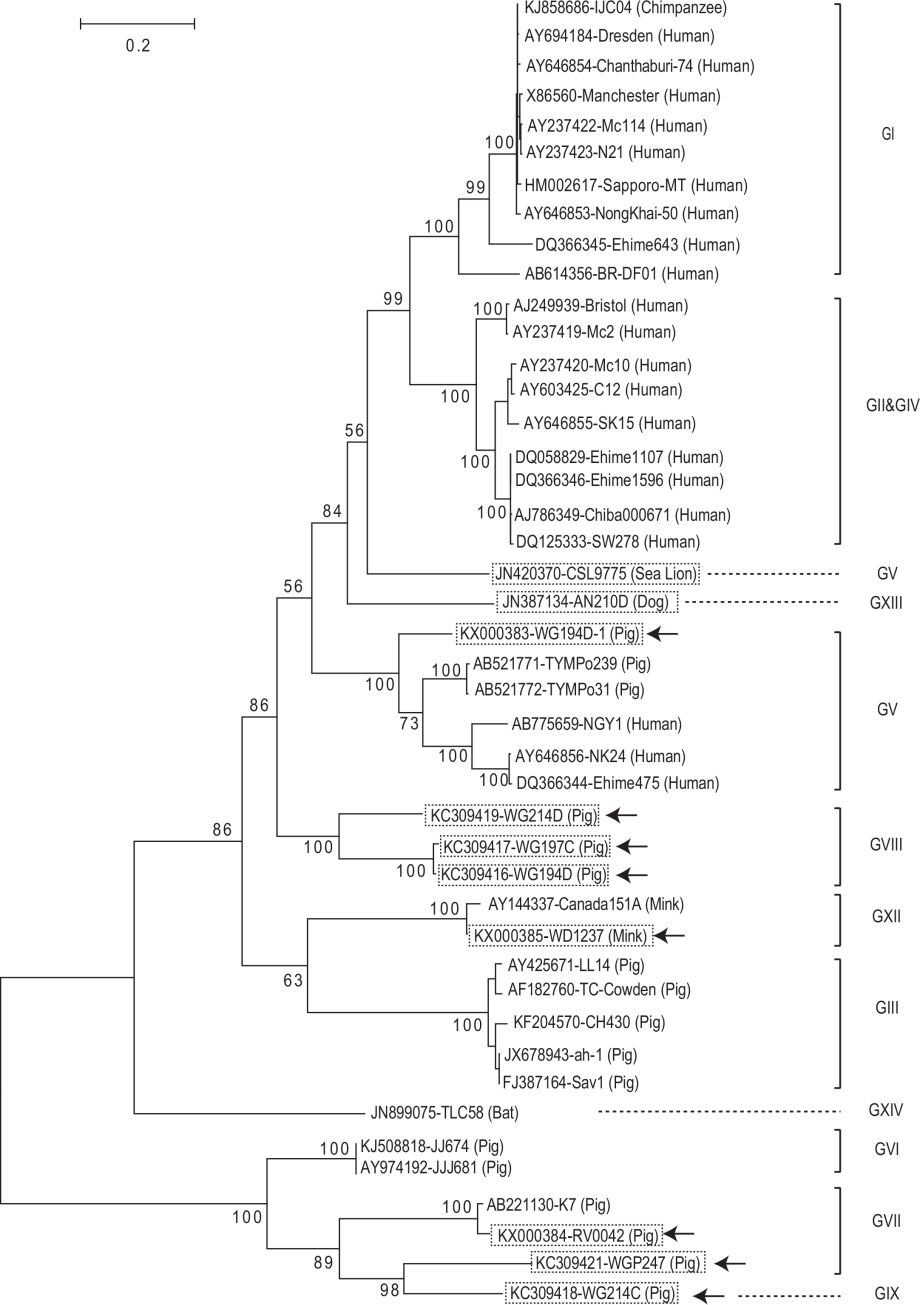
Phylogenetic tree of the putative NS7 amino acid sequences (approx. 500 aa) of sapovirus strains. These 44 strains represent 12 genogroups (GI-GV, GVI, GVII, GVIII, GIX, GXII, GXIII, and GXIV) using MEGA 6. The amino acid sequences covering from “WKGL” sequence to the end of the putative NS7, “XXME”, were used. Only 44 of the 74 SaV strains in the VP1-tree (Fig 3) have sequences for this region. The 10 strains analyzed in this study are boxed with dotted lines. Among these, the eight SaV strains whose corresponding sequences were determined in this study are indicated with arrows. The NS7 sequences of porcine GIX and GX SaVs, and rat GII and GXV SaVs are not yet available. The number on each branch indicates the bootstrap value. The scale represents the amino acid substitutions per site. Each sapovirus strain is indicated in the following format: Genbank accession number-strain name (species).

From a different perspective, we noted that the first five amino acid sequences (M [A or V] S [K or R] P) of the putative NS1 and the five amino acid sequences surrounding the putative cleavage site between NS7 and VP1 (FEME/G: slash is the putative cleavage site) [23] of porcine GV and GVIII, sea lion GV and dog GXIII SaVs were similar to those of GI, GII, GIV, and GV human SaVs (M[A or V] S [K or R] P and FEME/G, respectively). In contrast, these sequences are clearly different in porcine GIII (M [A/ P] NCR and FVME/A), GVI (MAATC and YTMEG), GVII (MAA [V/T] C and Y [K/V] MEG), and bat GXIV SaVs (MAALS and FVMEG) (Table 1).

From these observations we can infer that some animal SaVs (porcine GIII, GVI and GVII and bat GXIV) evolved more distantly from human SaVs than others (porcine GV and GVIII, sea lion GV and dog GXIII). We compared such amino acid sequence characteristics partially for GIX and GXII SaVs. Only the putative cleavage sites between NS7 and VP1, YVMEG and FEMEG of GIX and GXII SaVs, respectively, are available. We could not do a similar analysis for porcine GX and GXI, and the rat GII and GXV SaVs, because the corresponding amino acid sequences are not available (Table 1).

## Conclusions

Using the long RT-PCR strategy with our newly designed forward primer targeting the conserved region of the putative NS3 and the 5′ RACE methods, we identified and/or determined additional complete genomic sequences for GV, GVII, and GVIII SaVs. We also characterized the genomic extremes of sea lion GV and canine GXIII SaVs. Further determination of animal SaV genome sequences, including those SaVs (porcine GIX, GX and GXI, mink GXII, and rat GII and GXV), whose complete genomes have not been determined, may enable the design of more universal SaV primers to detect SaVs from both animals and humans. In future studies it will also be interesting to evaluate the potential interspecies transmission of closely related animal and human SaVs, such as GI, GII and GV SaVs, using experimental animals.
